# Resonant microwave fields and negative magnetic response, induced by displacement currents in dielectric rings: theory and the first experiments

**DOI:** 10.1038/s41598-017-02310-1

**Published:** 2017-05-19

**Authors:** A. B. Shvartsburg, V. Ya. Pecherkin, L. M. Vasilyak, S. P. Vetchinin, V. E. Fortov

**Affiliations:** Joint Institute for High Temperatures, Russian Academy of Sciences, Moscow, 125412 Russia

## Abstract

The theoretical basis and experimental verification of resonant phenomena in the electromagnetic fields generated by displacement current in the near zone of dielectric ring is presented. According to the traditional viewpoint, the dielectric has an influence on the electric field inside resonator. To the contrary, we demonstrate that the dielectric ring exhibits magnetic properties at resonance. The sliding incidence of plane microwave on this weakly absorbing ring is shown to provide the sharp and deep resonance in the components of generated field; this low loss circuit is operating as a resonant dielectric magnetic dipole. Splitting and broadening of resonance in the pair of these dipoles dependent upon their mutual arrangement is recorded. The phase shift equal to *π* between the magnetic components of incident and generated wave indicating the formation of negative magnetic response is demonstrated. Perspectives of using of this simple sub wavelength resonant magnetic dipoles in the all-dielectric circuitry are discussed.

## Introduction

This paper is devoted to the unusual resonant properties of electric and magnetic fields generated in the vicinity of dielectric rings due to sliding incidence of electromagnetic waves on these rings. The self oscillations of displacement currents and magnetic fields in these rings can be compared with the resonant effects in the first oscillating circuit, pioneered by W. Thomson as long ago as in 1853 y. This simple device, containing the inductance coil (self-inductance *L*), capacitor (capacity *C*) and the electric battery proved to be a prototype for numerous resonant *LC* filters and generators in the developing radio technique and electronics. Electrodynamics of displacement currents opens the new venues to creation of dielectric elements with predesigned electric and magnetic dipolar resonances. The possibilities to excite these resonances due to Mie scattering of electromagnetic waves on dielectric nanospheres^1–3^ and, in particular, on silicon^4^ and germanium^5^ nanospheres, had stimulated the growing interest to optically induced magnetization in dielectric media in the THz and IR spectral ranges. The perspectives to diversify these resonant effects are based on the controlled flexibility of electromagnetic parameters of dielectric metamaterials^6,7^. Special attention is given to so-called single-negative metamaterials, characterized by negative magnetic permeability (*μ* < 0) in some spectral ranges^8,9^. Pendry *et al*.^10^ pointed out, that such metamaterial can be designed by a kind of conductive horse-shoe-like circuits with splits (SRR), possessing the classical *LC*- resonance; two years later it was experimentally realized by Shelby *et al*.^11^. These results, obtained for microwaves, were rescaled for conductive currents in the nanoscale SRR operating in the THz range^12,13^. Using of *LC*- resonance in structures containing the spherical metallic inductance elements was considered in refs^14^,^15^ and golden rectangular micro-rod^16^. To the contrary, the examples of inductance excited by displacement currents in non-metallic elements and providing the formation of resonant frequencies were calculated for all-dielectric cylinders^17^ and rings^18^. Using of displacement currents in dielectric structures opens the possibility to combine the inductive and capacitive properties in one element.

This research is aimed at the theoretical analysis and experimental verifications of resonant effects in the excitement of electromagnetic fields in the near zones of dielectric rings, irradiated by plane microwaves; resonance in the electric components of excited fields and their phase shifts will be demonstrated. This work is different from works elaborating split-ring metallic resonator structures since it is devoted to non-split all-dielectric circuit. Unlike the resonance in split-ring resonators, stipulated by conductivity currents, resonance in dielectric rings is linked with the displacement currents. Note, that the theoretical analysis of Mie scattering on cylinders and spheres is based usually on the well known solutions of wave equations for these scatterers; since such solutions for the ring-shaped scatterer are unknown, the problem under discussion is treated below from the first principles based on the Faraday electromagnetic inductance.

**Figure 1:**
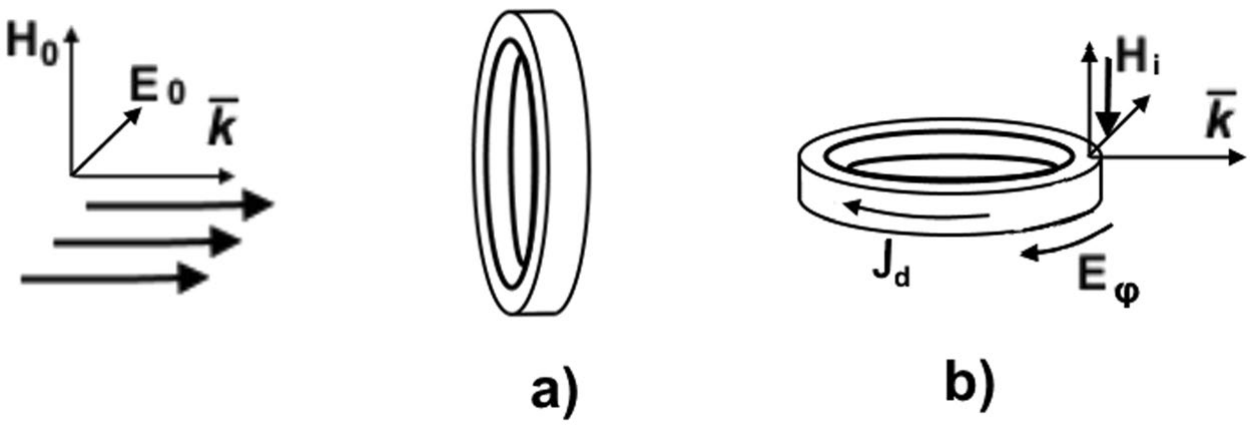
Geometry of the problem. (**a**) Polarization structure of incident wave; plane of the ring is perpendicular to the wave vector $\vec{k}$. (**b**) Plane of the ring is perpendicular to the magnetic component of the incident wave $\vec{H}$. This arrangement is used for resonant excitement of electromagnetic oscillations in all-dielectric thin ring-shaped circuit due to sliding incidence of plane linearly-polarized electromagnetic wave on this ring; ${\vec{J}}_{d}$ - the azimuth displacement current excited in the ring, *E*_*φ*_ - the azimuth electric field excited in the ring.

The ring with circular current can be viewed as a magnetic dipole. Consideration of magnetic dipoles in the microwave range is linked usually with the design of frame aerials and their radiation pattern in the far zone^19^. To the contrary we’ll investigate the microwave field in the near zone of magnetic dipole, formed by displacement current-carrying dielectric ring, and it’s resonant properties stipulated by it’s finite sizes. Let us examine the interaction of electromagnetic wave with a thin torus-like dielectric ring; the big and small radii of this ring, respectively *R* and *r*_0_, obey to condition $R\gg {r}_{0}$. The plane linearly polarized monochromatic wave with frequency *ω* and wave number *k* incidents on this ring in *z*-direction; the ring’s diameter is less than the wavelength. The wave’s magnetic field $H={H}_{0}\,\exp \,[i(kz-\omega t)]$ and electric field *E* are directed along the *x*-axis, normal to (*z*, *y*) plane, and y -axis respectively. To compare and contrast our analysis with previously known values of some resonant frequencies of such ring we’ll examine two different arrangements of this ring with respect to the incident wave beam. In case I the ring is located in a plane normal to the wave vector $\vec{k}$ of the incident wave beam (Fig. 1a). To the contrary, in case II the ring’s plane is perpendicular to the magnetic vector $\vec{H}$ and parallel to the wave vector $\vec{k}$ of the incident wave (Fig. 1b).

In case I the lowest resonant frequency of the ring viewed as a piece of coaxial cavity can be calculated by means of results^19^; to test our measuring system this resonance was confirmed in the experiment. After this verification our research was focused on another resonance arising in case II. The wave field *H* in this geometry forms the alternating magnetic flow Φ_0_ exp (−*iωt*) passing through the ring. Owing to the electromagnetic induction this flow is generating the electromotive force *U* in the ring and the azimuthally directed current *I*, which, in its turn, generates the secondary flow Φ and alternating electromagnetic field. The flow Φ is distinguished from Φ_0_ exp (−*iωt*) due to self-inductance of the ring.

It is noticeable, that the temporal and spectral dependences of the induced circular currents *I* are different in cases of conducting and non-conducting rings, the radii of the rings and the initial flow Φ_0_ exp (−*iωt*) being the same. Thus, in case of conducting ring the values of induced electromotive force *U* and conduction current *I* = *I*_*c*_ are

1 $$U=-\frac{1}{c}\frac{\partial {\rm{\Phi }}}{\partial t};\,{I}_{c}=\frac{U}{Z};$$

here *Z* is the impedance of the ring; thus, the current *I*_*c*_ is proportional to the ***first*** temporal derivative of magnetic flow Φ.

To the contrary, the electromotive force *U* determined by the first formula in (1) generates inside the non-conducting ring the azimuthally directed electric field *E*_*ϕ*_

2 $${E}_{\phi }=\frac{U}{2\pi R};$$

This field *E*_*ϕ*_ located in the non-conducting ring with dielectric permittivity *ε* forms the electric displacement *D* = *εE*_*ϕ*_; this alternating displacement stipulates the generation of circular displacement current *I*_*d*_ in the ring^20^

3 $${I}_{d}=\frac{{S}_{0}}{4\pi }\frac{\partial (\varepsilon {E}_{\phi })}{\partial t};$$

Here ${S}_{0}=\pi {r}_{0}^{2}$ is the area of cross-section of the ring (Fig. 1). Inspection of equations (1) and (3) shows, that the current *I*_*d*_ is proportional to the ***second*** temporal derivative of magnetic flow Φ. This distinction is shown below to provide the fundamental differences in spectral properties and magnetic response of induced electromagnetic fields.

The paper is organized as follows: the theoretical spectra of resonant circular curl electric field in the vicinity of a single sub wavelength dielectric ring, induced by the displacement current *I*_*d*_ in the ring, are presented in Chapter 2; the resonant phase shift between the magnetic components of incident and induced fields, close to *π* and indicating the formation of negative magnetic response of this oscillating circuit is considered. Classical Thomson *LC* circuits and the dielectric rings, viewed as the magnetic dipoles, are compared in Chapter 3; interaction of displacement currents in a pair of magnetically coupled rings, is shown to provide the splitting of resonant frequencies. The experiments verifying these theoretical results are described in Chapter 4. Some physical peculiarities and eventual applications of this all-dielectric circuitry are listed in Chapter 5.

### Resonant excitement of electromagnetic fields by displacement current - carrying rings

To examine the electromagnetic fields, excited by current – carrying ring, one can use the vector-potential, created by this current *I* in any observation point *O*_1_. The azimuthally directed component of vector-potential *A*_*ϕ*_ can be written in a form ref.^20^

4 $${A}_{\phi }=\frac{2IR}{c}\aleph ;\,\aleph ={\int }_{0}^{\pi }\frac{\exp \,(ikr)}{r}\,\cos \,\phi d\phi ;$$

here *r* is the distance between the observation point *O*_1_ and the ring centre *O*, *k* is the wave number of excited field, *ϕ* is the azimuth angle in the plane of ring. The components of electromagnetic field induced by current *I* outside the ring are expressed via *A*_*ϕ*_^20^

5 $${E}_{curl}=-\frac{1}{c}\frac{\partial {A}_{\phi }}{\partial t};\,{H}_{x}=\frac{\partial {A}_{\phi }}{\partial \rho }+\frac{{A}_{\phi }}{\rho };\,{H}_{\rho }=-\frac{\partial {A}_{\phi }}{\partial x};$$

The current *I* in (4) is determined, in its turn, by the magnetic flow Φ passing through the ring in the plane *x* = 0. To find this flow one has to subtract the inductance flows Φ_*i*_ and Φ_*c*_ generated respectively by displacement and conductive currents *I*_*d*_ and *I*_*c*_ in the ring with self-inductance *L* from the initial flow Φ_0_ exp (−*iωt*), generated by the magnetic component of incident wave:

6 $${\rm{\Phi }}={{\rm{\Phi }}}_{0}\,\exp \,(-i\omega t)-{{\rm{\Phi }}}_{i}-{{\rm{\Phi }}}_{c};$$

7 $${{\rm{\Phi }}}_{0}={H}_{0}F;\,F=\int \exp \,(ikz)\,dS;\,F=\pi {R}^{2}f\,(kR)\,\exp \,(ikR);\,f\,(kR)=\frac{2{J}_{1}\,(kR)}{kR};$$

here *J*_1_(*x*) is the Bessel function of the first kind. For more details see supplementary information. In a long-wave limit $kR\ll 1$ one gets *f*(*kR*) → 1, and the expression for Φ_0_ (A3) is reduced to the obvious result: Φ_0_ = *H*_0_ *πR* ^2^.

8 $${{\rm{\Phi }}}_{i,c}=\frac{L{I}_{d,c}}{c};\,L=4\pi R{l}_{1};\,{l}_{1}=\,\mathrm{ln}\,(\frac{8R}{r})-\frac{7}{4};$$

The currents *I*_*c*_ and *I*_*d*_ are determined in eqs (1) and (3), herein the quantity *Z* in (1) presents the active resistance of the ring fabricated from material with conductivity *σ*: *Z* = 2*R*(*r* ^2^ *σ*)^−1^. When eqs (1)–(3) and (8) are plugged into eq. (6), one gets:

9 $$\frac{{\partial }^{2}{\rm{\Phi }}}{\partial {t}^{2}}+\gamma \frac{\partial {\rm{\Phi }}}{\partial t}+{\omega }_{0}^{2}{\rm{\Phi }}={\omega }_{0}^{2}{{\rm{\Phi }}}_{0}\,\exp \,(-i\omega t);$$

10 $${\omega }_{0}^{2}=\frac{2{c}^{2}}{{r}^{2}\varepsilon {l}_{1}};\,\gamma =\frac{4\pi \sigma }{\varepsilon };$$

here *ω*_0_ and *γ* are respectively the characteristic frequency and attenuation constant of magnetic flow oscillations. Solution of eq. (9) presenting the magnetic flow Φ reads as

11 $${\rm{\Phi }}={{\rm{\Phi }}}_{0}{\rm{\Lambda }}\,(\omega )\,\exp \,(-i\omega t);\,{\rm{\Lambda }}\,(\omega )=\frac{{\omega }_{0}^{2}}{{\omega }_{0}^{2}-{\omega }^{2}-i\omega \gamma };$$

here Λ(*ω*) is the dimensionless resonant factor. Substitution of solution (11) to eqs (1)–(3) brings the displacement current in the ring *I*_*d*_:

12 $${I}_{d}=\frac{{S}_{0}\varepsilon {\omega }^{2}{{\rm{\Phi }}}_{0}{\rm{\Lambda }}\,(\omega )\,\exp \,(-i\omega t)}{8{\pi }^{2}Rc};$$

the forthcoming substitution of *I*_*d*_ to eqs (4) and (5) yields the vector-potential *A*_*ϕ*_ and the components of induced electromagnetic field. Thus, using the expression for magnetic flow Φ_0_, we obtain the normalized value for circular electric field *E*_*curl*_, induced by current *I*_*d*_ outside the ring:

13 $$\frac{{E}_{curl}}{{H}_{0}}=-\frac{i{\omega }^{3}\varepsilon {r}^{2}R\aleph {\rm{\Lambda }}\,(\omega )\,f\,\exp \,[i(kR-\omega t)]}{4{c}^{3}};$$

One can see from eqs (9)–(13), that the excitement of electric currents and fields possesses the resonant properties, herein the resonant frequencies in the cases, shown on Fig. 1a,b, are distinguished drastically: thus, the evaluations for the given sizes of rings (see Chapter 4) yield the resonant frequencies 15 GHz for case 1a, widely used in the microwave technique, and 1.32 GHz for case 1b, discussed here.

The expressions for magnetic components of induced field can be calculated from (5) on the same way. The dimensionless factor $\aleph$ (4) contains the information about the spatial structure of field components. Omitting here the analysis of this spatial structure let us stress out the common spectral property of field components described by the resonant factor Λ(*ω*). This factor, determining the change of sign for both electric and magnetic components due to frequency transition through the resonant value $\omega \approx {\omega }_{0}$, indicates the reverse of directions of these components to the opposite ones; herein, the directions of components of incident wave remain unchanged. In particular, the resonant factor Λ(*ω*) reveals the important property of induced magnetic field *H*_*x*_: transition of frequency from the spectral range *ω* < *ω*_0_ to the range *ω* > *ω*_0_ stipulates the reverse of induced field component *H*_*x*_ with respect to the incident wave field *H*; this change is accompanied by the phase shift of *H*_*x*_ close to *π*. The reverse of magnetic field *H*_*x*_ can be viewed as a negative magnetic response of this resonant magnetic dipole. The phase shift between the electric components of incident and induced fields indicating the formation of negative magnetic response, verified experimentally, is presented in Chapter 4. Herein, the displacement current-carrying ring can be considered as a resonant all-dielectric magnetic dipole with the magnetic moment $\vec{m}={I}_{d}\pi {R}^{2}\vec{n}$; here vector $\vec{n}({|\vec{n}|}^{2}=1)$ is perpendicular to the ring’s plane. These dipoles may become perspective for using as the unit cells of singular magnetic metamaterials, characterized by negative magnetic permeability and positive dielectric susceptibility^15^.

### Dielectric magnetic dipoles vs Thomson oscillating circuits

It is noteworthy to stress out the analogy between the dielectric ring under discussion and the classical Thomson *LC* oscillating circuit: if we express the small radius of the ring *r*_0_ and the quantity *l*_1_ via the area ${S}_{0}=\pi {r}_{0}^{2}$ and ring self-inductance *L* (8) respectively, the formula for resonant frequency *ω*_0_ (10) will coincide with the classical Thomson formula for resonant frequency of the oscillating circuit containing the self-inductance *L* and capacitance *C*: ${\omega }_{0}=c/\sqrt{LC}$; herein the quantity $C=\varepsilon {S}_{0}{(8{\pi }^{2}R)}^{-1}$ can be viewed as a capacitance of some fictitious plane capacitor $C=\varepsilon S{(4\pi D)}^{-1}$, where the areas of plates *S* and the distance between them *D* are equal respectively to the area *S*_0_ and the length of ring’s circumference *D* = 2*πR*. Thus, the dielectric ring (Fig. 1b) can be viewed as an oscillating circuit, possessing simultaneously the properties of both capacitor and inductor.

Using this analogy one can find the resonant frequency of split thin dielectric ring, containing two arcs, separated by narrow slits with width *d*. Considering these arcs and slits as the capacitors arranged in series configuration one can find the resonant frequency of this *LC* circuit. Each slit can be viewed as a plane capacitor formed by the plates with area *S*_0_ spaced by distance *d*; taking into account the length *s* = *πR* − *d* of each of two arcs, forming the split circumference, we obtain for the capacity *C*_1_ of chain containing two arcs and two slits

$$\frac{1}{{C}_{1}}=\frac{8{\pi }^{2}R}{\varepsilon {S}_{0}}[1+\frac{d}{\pi R}\,(\varepsilon -1)];$$

Substitution of this value *C*_1_ to Thomson’s formula yields the resonant frequency of split ring ${\rm{\Omega }}$; in a case $\varepsilon \gg 1$ we have

14 $${\rm{\Omega }}={\omega }_{0}\sqrt{1+2\varepsilon \kappa };\,\kappa =\frac{d}{2\pi R}\ll 1;$$

The shift of resonant frequency stipulated by these slits is shown below in Chapter 4. Proceeding in a similar fashion one can find the resonant frequency of a split dielectric ring with one slit ${{\rm{\Omega }}}_{1}={\omega }_{0}\sqrt{1+\varepsilon \kappa }$. This circuit resembles the SRR, pioneered by Pendry *et al*.^10^; however, unlike the metallic split ring with inevitable ohmic losses^11^, the losses in the all-dielectric ring are essentially smaller.

The approach, based in the *LC* circuit model, can be generalized for the system of magnetically coupled dielectric rings; the coupling is based on the interference of their magnetic flows. This interference can be characterized by the coefficients of mutual inductance *M* of displacement currents excited in the rings. To stress out the salient features of this interference consider the pair of similar thin rings with equal radii *R*. Coefficient *M* is known to depend upon the mutual arrangement of rings: thus in a simple case of coaxial rings, spaced by distance *b*, coefficient *M* reads as ref.^20^:

15 $$M=4\pi R{l}_{2};\,{l}_{2}=\frac{2\wp \,(q)}{q};\,\wp \,(q)=(1-\frac{{q}^{2}}{2})K(q)-E(q);\,{q}^{2}=\frac{1}{1+{\eta }^{2}};\,\eta =\frac{b}{2R};$$

here *K*(*q*) and *E*(*q*) are respectively the complete elliptic integrands of the first and second kind^21^ with modulus *q*.

The magnetic flows (Φ_*i*_)_1,2_ excited in these rings by displacement currents *I*_1,2_ due to self-inductance and inductance effects can be written as

16 $${({{\rm{\Phi }}}_{i})}_{1,2}=\frac{1}{c}(L{I}_{1,2}+M{I}_{2,1});$$

Designating the magnetic flows formed by the incident wave in these rings as Φ_01_ exp (−*iωt*) and Φ_02_ exp (−*iωt*) and neglecting the dissipation effects (*γ* → 0) one can write the set of equations governing the resulting magnetic flows Φ_1_ and Φ_2_ passing through these rings

17 $$\frac{1}{{\omega }_{1}^{2}}\frac{{\partial }^{2}{{\rm{\Phi }}}_{1}}{\partial {t}^{2}}+\frac{1}{{\omega }_{2}^{2}}\frac{{\partial }^{2}{{\rm{\Phi }}}_{2}}{\partial {t}^{2}}+{{\rm{\Phi }}}_{1}={{\rm{\Phi }}}_{01}\,\exp \,(-i\omega t);$$

18 $$\frac{1}{{\omega }_{1}^{2}}\frac{{\partial }^{2}{{\rm{\Phi }}}_{2}}{\partial {t}^{2}}+\frac{1}{{\omega }_{2}^{2}}\frac{{\partial }^{2}{{\rm{\Phi }}}_{1}}{\partial {t}^{2}}+{{\rm{\Phi }}}_{2}={{\rm{\Phi }}}_{02}\,\exp \,(-i\omega t);$$

Here the characteristic frequencies for similar coaxial dielectric rings linked with the self inductance of each ring *ω*_1_ and mutual inductance of rings *ω*_2_ are

19 $${\omega }_{1,2}=\frac{c\sqrt{2}}{r\sqrt{\varepsilon {l}_{1,2}}};$$

Dimensionless quantities *l*_1_ and *l*_2_ are determined in (8) and (15) respectively. The resonant frequencies of coupled electromagnetic oscillations for the pair of these rings found from eqs (17) and (18) are

20 $${\omega }_{\pm }=\frac{{\omega }_{1}}{\sqrt{1\pm \tfrac{{l}_{2}}{{l}_{1}}}};$$

Thus the coupling between the dielectric magnetic dipoles results in splitting of their spectra. Note, that in case of strong mutual inductance (*l*_2_ > *l*_1_) only one mode with *ω*_+_ does exist; in this case the resonance proves to be not splintered, but shifted. In a limiting case, when the effect of magnetic coupling becomes negligible (*l*_2_ → 0), the splitting is vanishing: *ω*_±_ → *ω*_1_.

### Experimental microwave spectra of resonant magnetic dipoles

**Figure 2:**
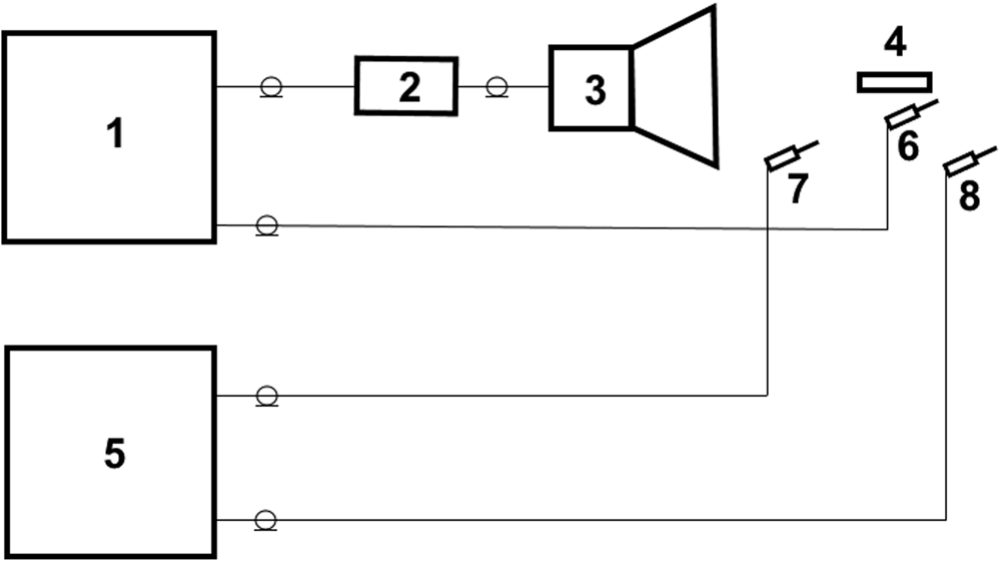
Experimental setup scheme. (**1**) Network analyzer. (**2**) RF broadband amplifier. (**3**) Double ridged waveguide horn antenna. (**4**) Dielectric ring. (**5**) Four channel oscilloscope. (**6**) High frequency probe of network analyzer. (**7**) Reference high frequency probe of oscilloscope. (**8**) High frequency probe of oscilloscope.

The measurements of electric field, generated by the displacement current in the ring, were performed by means of the setup, shown schematically in Fig. 2.

**Figure 3:**
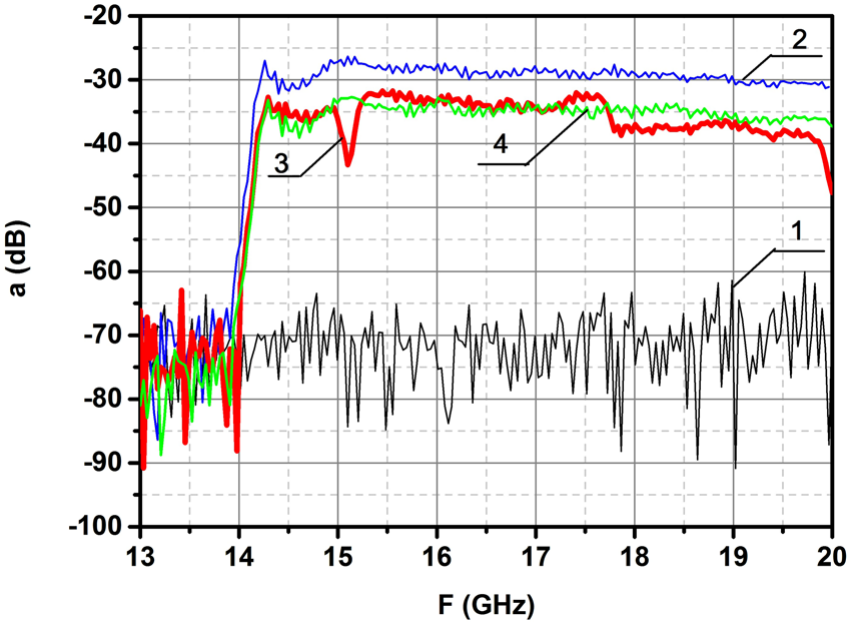
Experimental spectra for the arrangement of dielectric ring, depicted in Fig. 1a. The distance between antennas is 30 cm. Spectra **1**, **2**, **3** show the level of noises, the level of radiation without the ring and level of radiation in the centre of ring respectively. The resonant dip at the frequency *ω*_0_ = 15.1 GHz is recorded. To the contrary, the spectrum of brass ring (curve **4**) doesn’t display the resonant behaviour.

The results of spectral measurements for the arrangement of dielectric ring, shown in Fig. 1a, are presented in Fig. 3.

**Figure 4:**
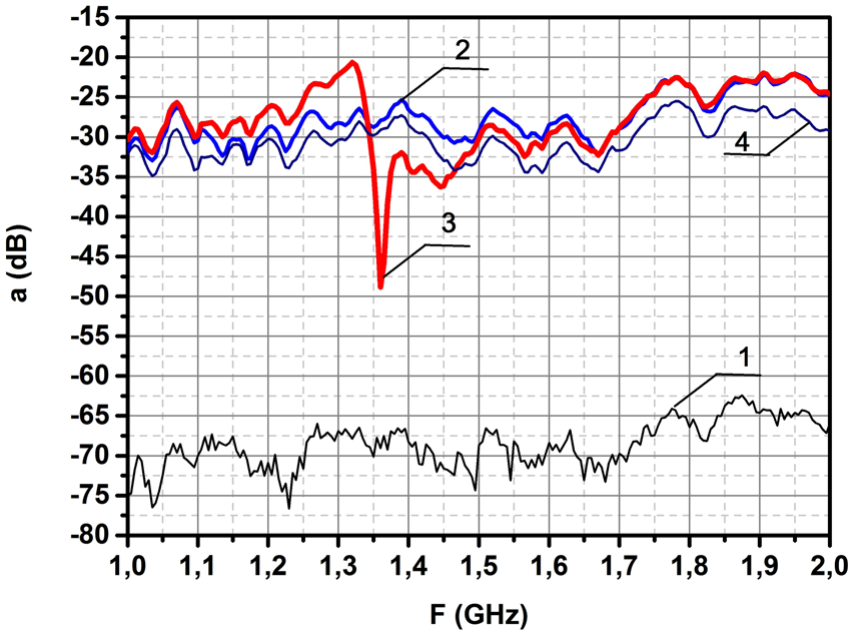
Measured spectra of azimuthally directed induced electric field *E*_*curl*_ outside of dielectric ring, depicted in Fig. 1b. The distance between antenna and probe is 60 cm. Spectra 1, 2, 3 show the level of noises, the level of radiation without the ring and the level of radiation in the centre of ring respectively. The resonant dip at the frequency *ω*_0_ = 1.36 GHz is recorded. To the contrary, the spectra of brass ring (curve 4) doesn’t display the resonant behaviour.

Observation of scattering on this ring in the spectral range 12.4–20 GHz had revealed the resonant frequency *f* = 15.1 GHz. This figure is in good agreement with the value *f* calculated for this geometry in the framework of theory of dielectric coaxial cavities^19^. Thus, this measurement, carried out in order to control the experimental system, brought the well known result. Note, that irradiation of the brass ring with the same sizes didn’t indicate any resonance in this range (curve 4). To the contrary, the spectral measurements in the range of 1–2 GHz for case II (Fig. 1b) had revealed the novel resonance at the frequency *f*_0_ = 1.36 GHz (Fig. 4 curve 3). Experimental value of resonant frequency is in good agreement with the theoretical value found in Chapter 2. Several oscillation modes can be excited in the dielectric rings. Frequency 15.1 GHz is linked with the azimuth oscillations excited by the electrical component of incident wave; these oscillations are widely used in the series of microwave devices. To the contrary the frequency 1.36 GHz is excited by the magnetic component of incident wave. Herein the half bandwidth of this resonance Δ = 20 MHz indicates the low losses in the dielectric ring, ensuring the possibility to use this effect in creation of new single-negative materials. Note, that in the metallic split-ring resonators such low losses are impossible^10,11^.

To compare this result with the theoretical value of resonant frequency (10), dependent upon the self-inductance factor *l* (8), one has to recall, that this formula relates to the current-carrying ring with circular cross-section (radius *r*). This formula, describing the magnetic interaction of different parts of current-carrying ring with the circular cross-section (radius *r*_0_), is derived^20^ due to integration of some expression, divergent for the infinitesimal distances between these parts. To avoid this divergence, the value *r*_0_ is taken in (8) as a smallest distance discussed. However, in our experiment we are dealing with the thin ring with square cross-section. Keeping in mind the logarithmic accuracy of formula (8) for *l*, one can use, instead of *r*_0_, some small scale, habitual to square cross-section, e.g., the size of square side *a*. Herein, the replacement of values *r*_0_ → 0.5*a*, *R* → *R*_*eff*_ = *R* − 0.5*a* in (10), brings the resonant frequency *f*_0_ = 1.32 GHz, close to the experimental value. Side by side with the rings arrangements, shown on the Fig. 1b,c, the scattering spectrum of dielectric ring disposed perpendicularly to the vector $\vec{E}$ of incident wave, was measured in the spectral range 1–20 GHz; herein the wave’s magnetic component ${\vec{H}}_{0}$ was located in the ring’s plane. No resonant frequency in this geometry was recorded; this result visualizes again the dominant role of displacement current induced in the ring in the geometry shown on Fig. 2b. Note, that these experiments were carried out for the stationary rings. The rings rotation can stipulate the series of interesting non – stationary phenomena^21,22^.

**Figure 5:**
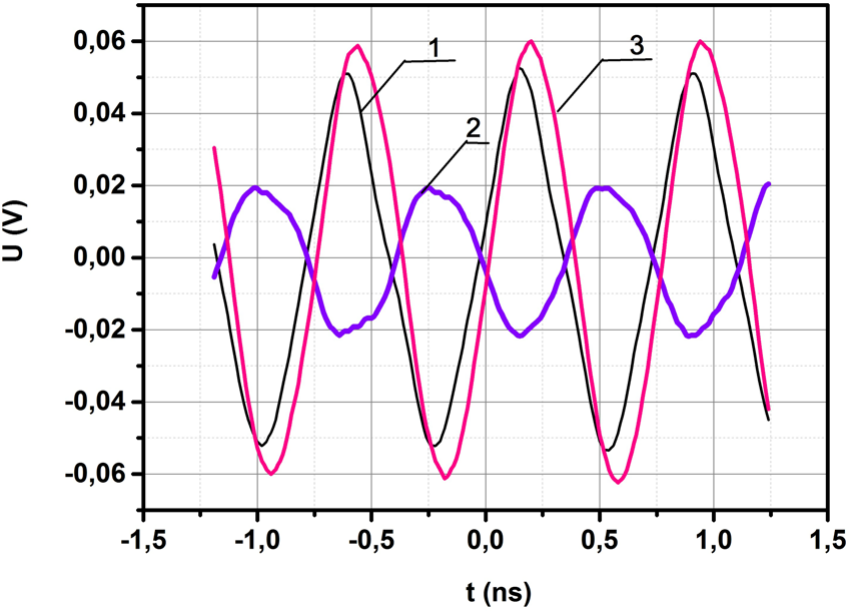
Waveforms of electric field component near the dielectric ring. (**1**) Reference probe. (**2**) Second probe near the back side of dielectric ring kept away from antenna. (**3**) Second probe near the front side of dielectric ring to antenna.

An another effect of resonant scattering of incident wave is known to be linked with the phase shift of scattered wave, close to *π*, due to frequency transition through the resonant value. This effect is accompanied by the aforesaid reverse of directions of both electric and magnetic components of scattered field, displayed by the change of sign of resonant factor Δ(*ω*) (11) in the vicinity of resonant frequency *ω* = *ω*_0_, providing the attenuation in the circuit to be weak. The obtained results are shown in Fig. 5. In the course of ring’s displacement with respect to the second probe the phase shift close to *π* between signals from the ring and the referent probe was detected near the far side of the dielectric ring from the antenna (curve 2), indicating the formation of negative magnetic response of dielectric ring with displacement current. It should be noted that the amplitude of the signal from the probe, which is located near the front side of the ring towards the antenna (curve 3) is higher than the amplitude of the signal from the reference probe (curve 1) without a ring, due to the interference of incident and reflected waves.

An extremely important issue for this problem is the influence of the shape of the dielectric object on the observed resonance phenomena, either the different shapes of dielectric scatterers, providing their volumes to be equal, will cause their distinguished resonant frequencies? To compare and contrast these shape-dependent resonances one can recall the oscillating spectra of displacement currents induced, e.g., in the cylinder and ring. In accordance with ref.^19^ the difference in their resonant frequencies can be illustrated due to analysis of wave propagation in the transmission lines, formed, respectively, by the circular waveguide and coaxial line, since the different types of modes, existing in these lines, are well known. Thus, the coaxial waveguide supports the transversal TEM mode, meanwhile the modes in the cylindrical waveguide have the longitudinal field components. Owing to the distinctions in the polarization structure of propagating modes, stipulated by the geometry of these transmission lines, the resonant frequencies for the ring and cylinder prove to be different.

**Figure 6:**
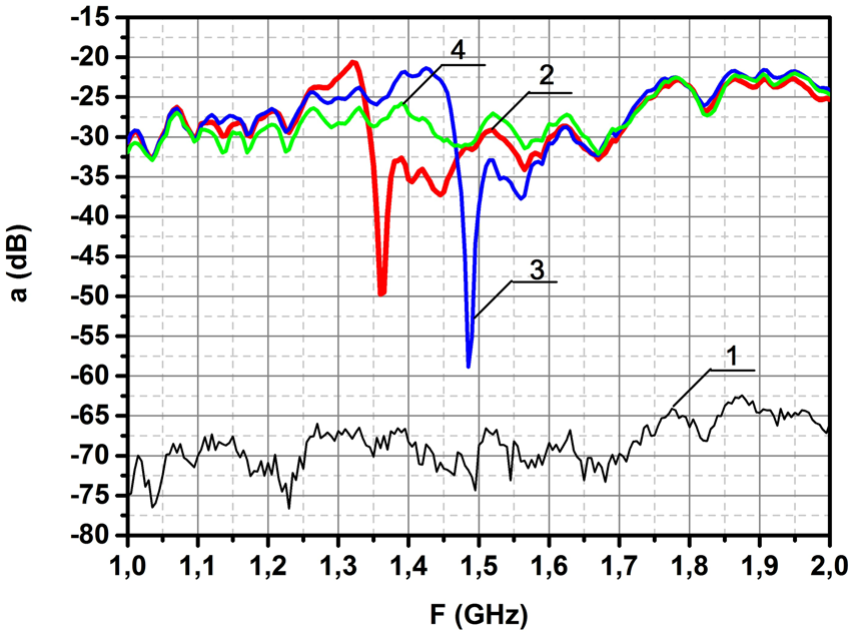
Shift of resonant frequency for the split dielectric ring. (**1**) Noise level. (**2**) Resonant dip of a whole ring. (**3**) Resonant dip for the split dielectric ring with the width of slit *d* = 0.01 cm. (**4**) Spectrum of horse shoe-like scatterer with the doubled thickness formed from two halves of ring.

The dependence of resonances upon the geometrical parameters of scatterers was investigated in experiments with two scatterers characterized by equal volumes but different shapes. To visualize the difference in their scattering spectra the dielectric ring with resonant frequency 1.36 GHz, described above, was hacked on two equal halves. In one case both halves were composed together, forming one half ring with the double thickness, resembling the horse shoe; in an another case both halves were added, forming one ring with two narrow slits. The scattered electric fields were measured in both cases, the results of measurements are presented in Fig. 6. To compare and contrast the observed effects the spectrum of a safe ring, borrowed from Fig. 4, is presented here too (curve 2). In case of horse shoe-like scatterer no any resonant effect was found (curve 4), meanwhile the spectrum of split dielectric ring, consisting from two halves of the ring located in one plane and separated by narrow slits *d* = 0.01 cm, is characterized by some shift of resonant frequency (curve 3).

Substituting the value of slit thickness *d* = 0.01 cm to (14) we’ll find the resonant frequency of split ring: *f*_*s*_ = 1.18*f*_0_, indicating the shift *f*_*s*_ − *f*_0_ = 0.23 GHz. Note, that the sides of slit are not polished, therefore the “averaged” value of thickness *d* = 0.01 cm gives the rise to discrepancy between the calculated and measured frequency shifts.

## Conclusion

We have presented the theoretical basis and experimental demonstrations of resonant phenomena in the microwave electromagnetic fields, generated by displacement currents in the dielectric rings; these currents induced due to sliding incidence of linearly polarized microwave on the rings are shown to form the peculiar dielectric magnetic dipoles of finite sizes. The theory of these phenomena, generalizing the concepts of classical Thomson oscillating circuit, is elaborated from the first principles. Calculations of resonant frequencies of dielectric magnetic dipoles, their spectral splitting and shift are in good agreement with the experimental data. The main results of these researches are:

All-dielectric *LC* circuits are designed and tested in the GHz range. These circuits visualizing the spectral properties of lonely oscillating element ensure the possibility to model the near fields of nanoscale *LC* circuits inaccessible for the direct measurements now. Moreover, according to the traditional viewpoint, the dielectric has an influence on the electric field inside resonator. To the contrary, the theory and experiments, presented in our paper, stress out the resonant magnetic properties of dielectric rings. Up to our best knowledge, our paper presents the first manifestation of all-dielectric resonant ring-shaped magnetic dipoles, excited by displacement currents.

The resonant frequency of dielectric magnetic dipole dependent upon its sizes is calculated and measured. Formation of negative magnetic response in the resonant spectral band of this dipole is verified by means of phase measurements of electric fields, induced by the displacement current-carrying ring.

Splitting of resonances in spectra of these dielectric oscillators due to dipoles interaction and resonance shifts stipulated by slits in the rings are shown. Interference of fields generated by displacement currents in the pair of coupled rings opens the way to optimization of parameters of all-dielectric 3D systems of magnetic dipoles for the radiation control^23^.

The spatial structure of induced field *A*_*ϕ*_, determined by the dimensionless function $\aleph$(4), which isn’t calculated here, deserves the special attention. This structure has to be found, e.g., for consideration of the sizes of unit cell for single negative metamaterials. Although the analytical expressions for fields described by this function in the near $(kr\ll 1)$ and far $(kr\gg 1)$ zones are well known ref.^18^, the intermediate zone $(kr\approx 1)$ important for the design and optimization of these unit cells has to be examined numerically.

The important advantage of all-dielectric oscillating circuits is their insignificant absorption; thus in the microwave range the loss tangent is as small as 0.001. The practical usefulness of the obtained results is connected with an extremely small HF losses in dielectric structures as compared with the metallic ones. Due to smallness of losses the Q-factor of all-dielectric structures is increased and the width of resonance is narrowed. Design of resonant spectra for dielectric structures is more flexible, than the similar design for metallic ones, since the resonant frequency *ω*_0_ for dielectric ring depends upon both dielectric susceptibility and ring’s sizes, meanwhile the value *ω*_0_ for metallic structure depends upon its sizes only. This property paves the way to use the displacement currents in the low loss information-carrying systems. All-dielectric resonant magnetic dipoles, possessing the negative permeability, might represent a favorable alternative to traditional split-ring structures in design of bulk single-negative metamaterials.

## Methods

### Gigahertz Resonant Measurement

The experimental setup used for the verification of the theoretical results is shown in Fig. 2. The experiments were aimed at the excitation of lower resonant frequencies in the dielectric rings irradiated by the linearly polarized microwaves. Agilent E5071C ENA Network Analyzer was used for the generation and registration of emission spectra of GHz-range. Two different arrangements of the ring with respect to the electric $\vec{E}$ and magnetic $\vec{H}$ components of the incident wave were examined. In one case shown on Fig. 1a ETS-Lindgren’s model 3160-09 pyramidal horn antennas were employed as the transmitter and receiver. To weaken the influence of standing waves arising in the vicinity of the mouth of horn antenna^20^ the ring was shifted away from the mouth. The distance between antennas was 30 cm; the dielectric ring was disposed at the distance 12 cm from the receiving antenna. In the other case (Fig. 1b) ETS-Lindgren’s model 3115 double ridged waveguide horn antenna was applied as a transmitter. To increase the signal-to-noise ratio and decrease the influence of radio noise the additional amplifier was used (2, Fig. 2). The transmitter power was 10 mw. The linear probes of high frequency electric field with the length of sensitive element 1 cm (6–8, Fig. 2) were used for measurements of electric field near by the ring. The high frequency magnetic field in the near zone was registered by the screened circular probe with 0.5 cm diameter of sensitive element. Each ring with square cross-section used in these experiments is characterized by the value of dielectric permeability *ε* = 200; it’s external radius is *R* = 1.9 cm, thickness is *a* = 0.5 cm. The dielectric rings used in our experiments were fabricated from the HF capacitor ceramics characterized by loss tangent as small as 6 × 10^−4^ in the frequency range 3–10 GHz.

### Phase Shift Measurement

The study of the phase shift between the initial and the reradiated waves was performed using a high-speed four-channel oscilloscope Tektronix DPO73304DX (5, Fig. 2) with two probes of the electric field (7, 8, Fig. 2). One probe located near antenna beyond of the area of influence of dielectric ring was used as a referent one. Its signal was recorded by one channel of the oscillations detector. Another probe was located close to the ring. Its signal was fixed by the second channel. Both probes were oriented in the direction of maximum sensitivity to the electric field of the incident wave and the distance between the probes kept constant in order to avoid any supplementary phase shift between them in the measurement process. Phase shift between probes was observed by ring’s displacement with respect to the second probe in direction of vector $\vec{k}$ of the incident wave.

## Electronic supplementary material

Resonant microwave fields and negative magnetic response, induced by displacement currents in dielectric rings: theory and the first experiments
