# Erratum: Resonant microwave fields and negative magnetic response, induced by displacement currents in dielectric rings: theory and the first experiments

**DOI:** 10.1038/s41598-017-12313-7

**Published:** 2017-11-09

**Authors:** A. B. Shvartsburg, V. Ya. Pecherkin, L. M. Vasilyak, S. P. Vetchinin, V. E. Fortov

**Affiliations:** Joint Institute for High Temperatures, Russian Academy of Sciences, Moscow, 125412 Russia

*Scientific Reports* **7**:2180; doi:10.1038/s41598-017-02310-1; Article published online 19 May 2017

This Article contains typographical errors in the Acknowledgements section.

“This research was supported by the Russian Scientific Foundation under contract № 14-50-00124”.

should read:

“This research was supported by the Russian Scienсe Foundation (Project Number 14- 50-00124)”.

