# Supplementary material for: Resonant microwave fields and negative magnetic response, induced by displacement currents in dielectric rings: theory and the first experiments
Source: Sci Rep. 2017 May 19;7:2180. doi: 10.1038/s41598-017-02310-1 (PMC5438357; doi:10.1038/s41598-017-02310-1)
Supplement: Supplementary file 1 — Resonant microwave fields and negative magnetic response, induced by displacement currents in dielectric rings: theory and the first experiments [file 41598_2017_2310_MOESM1_ESM.pdf]

Resonant microwave fields and negative magnetic response, induced by displacement currents in dielectric rings: theory and the first experiments

A.B. Shvartsburg, V.Ya. Pecherkin*, L.M. Vasilyak, S.P. Vetchinin & V.E. Fortov

Joint Institute for High Temperatures, Russian Academy of Sciences, Moscow 125412, Russia,
*vpecherkin@ihed.ras.ru

Appendix 1

To obtain the expressions for magnetic flow Φ_0 in the explicit form one has to find the function F in formula (7). To calculate this function it is worthwhile to present the differential dS in (7) as the area of infinitesimal trapezium with the width dz [1]

$$dS = 2\sqrt{z(2R - z)}dz\,; \qquad \text{(A1)}$$

After the expression of coordinate z via the angle ϕ in the plane of ring $z = R(1 - \cos\phi)$, the differential dS (A1) reads as $dS = 2R^2 \sin^2 \phi d\phi$; using the integrand[2]

$$\int_0^\pi \cos(x\cos\phi)\sin^2\phi d\phi = \frac{\pi J_1(x)}{x}\,; \qquad \text{(A2)}$$

and bringing together (A1) and (A2) one gets

$$\Phi_0 = H_0 F\,; \quad F = \pi R^2 f(kR)\exp(ikR); \quad f(kR) = \frac{2J_1(kR)}{kR}\,; \qquad \text{(A3)}$$

here $J_1(x)$ is the Bessel function of the first kind. Substitution of (A3) to (4) yields the expression (13) for the electric component E_{curl} of the field induced in the near zone.

In a long-wave limit $kR << 1$ one gets $f(kR) \to 1$, and the expression for Φ_0 (7) is reduced to the obvious result: $\Phi_0 = H_0 \pi R^2$.

References

1. Sigov, A.S. & Shvartsburg, A.B. Microwave dielectric resonant oscillating circuit. *Doklady Physics* **61** № 7, 313-315 (2016).
2. Abramowitz, M. & Stegun, I. *Handbook of Mathematical Functions.* (Dover Publications, 1968).
